# Feedback control of combustion instabilities from within limit cycle oscillations using $H_{\infty }$ loop-shaping and the *ν*-gap metric

**DOI:** 10.1098/rspa.2015.0821

**Published:** 2016-07

**Authors:** Jingxuan Li, Aimee S. Morgans

**Affiliations:** Department of Mechanical Engineering, Imperial College London, London SW7 2AZ, UK

**Keywords:** combustion instabilities, flame describing function, *ℋ*_∞_ loop-shaping, *ν*-gap metric, level set approach, low-order controller

## Abstract

Combustion instabilities arise owing to a two-way coupling between acoustic waves and unsteady heat release. Oscillation amplitudes successively grow, until nonlinear effects cause saturation into limit cycle oscillations. Feedback control, in which an actuator modifies some combustor input in response to a sensor measurement, can suppress combustion instabilities. Linear feedback controllers are typically designed, using linear combustor models. However, when activated from within limit cycle, the linear model is invalid, and such controllers are not guaranteed to stabilize. This work develops a feedback control strategy guaranteed to stabilize from within limit cycle oscillations. A low-order model of a simple combustor, exhibiting the essential features of more complex systems, is presented. Linear plane acoustic wave modelling is combined with a weakly nonlinear describing function for the flame. The latter is determined numerically using a level set approach. Its implication is that the open-loop transfer function (OLTF) needed for controller design varies with oscillation level. The difference between the mean and the rest of the OLTFs is characterized using the *ν*-gap metric, providing the minimum required ‘robustness margin’ for an $H_{\infty }$ loop-shaping controller. Such controllers are designed and achieve stability both for linear fluctuations and from within limit cycle oscillations.

## 1. Introduction

It is desirable to operate modern industrial gas turbines and aeroengines under lean premixed combustion conditions in order to reduce NO_*x*_ emissions. However, lean premixed systems are highly susceptible to combustion instabilities arising from the coupling between the heat release rate perturbations and the acoustic disturbances within the combustion chamber [1]. These instabilities are nearly always undesirable as they lead to large oscillation amplitudes and damaging structural vibrations of the combustion chamber [2].

The stability of a combustion chamber is determined by the balance between the energy gained from the flame/acoustic interactions and various dissipation processes [1,3,4]. Active feedback control can be used to interrupt the coupling between the acoustic waves and unsteady heat release and prevent or suppress instability. The design of most types of controller requires prior knowledge of how the combustor responds to actuation—the ‘open-loop transfer function’ (OLTF) between monitored thermodynamic properties within the combustion chamber (typically one or more pressure measurements) and the actuator signal(s) [4].

Although linear models may be accurate at low perturbation levels, the dynamics of real unstable combustors become dominated by nonlinear mechanisms once amplitudes grow sufficiently. In many combustors, including gas turbine and aeroengine combustors, it is notable that even during large amplitude oscillations the behaviour of the acoustic waves remains linear, with nonlinearity arising almost entirely from the way in which the flame’s unsteady heat release responds to oncoming flow disturbances [5–7]. (Acoustic waves in rocket engines are, however, typically nonlinear [8].) This flame nonlinearity is ‘weak’ such that a ‘describing function’ provides a good model of the nonlinear flame response. This assumes that the flow forcing amplitude level is a quasi-steady parameter, and that the dominant frequency of the unsteady heat release rate response matches the incoming flow forcing frequency, but with a gain and phase shift that depend on the forcing level as well as the frequency. This gives rise to a ‘family’ of frequency responses that depend on forcing level. It has been shown that this weak nonlinearity can cause saturation into limit cycle either via saturation of the heat release rate amplitude or via a change in phase between the heat release rate and acoustic pressure as the modulation level increases [6,9]. This may cause the dominant unstable frequency to change, which, in turn, alters the OLTF and complicates the controller design. Although some recent work has identified nonlinear states other than limit cycle oscillations as resulting from combustion instability [10–12], the focus of this work will be limit cycle oscillations, which are by far the most common reported nonlinear state.

The design of linear feedback controllers is typically based on the OLTF corresponding to small, linear disturbances. Such feedback controllers are then activated from within nonlinear limit cycle oscillations, during which the linear OLTF will not be valid. It is typically observed that such controllers successfully stabilize oscillations despite this paradox [13–15]. However, their effectiveness is not guaranteed, and if they fail then there has, until now, been no framework for systematically improving their design.

In this work, the concept of a flame describing function (FDF) is combined with the *ν*-gap metric [16] in order to design controllers which are guaranteed to achieve stabilization across all oscillation levels, from small linear fluctuations to large limit cycle oscillations. In modern robust control systems theory, the concept of a *ν*-gap between open-loop plants represents the most useful measure of distance between systems when one is concerned specifically with applying feedback to those systems. The *ν*-gap metric fits very naturally into $H_{\infty }$ loop-shaping controller design, providing a bound on the minimum required ‘robustness stability margin’ for an $H_{\infty }$ loop-shaping controller [16,17]. Modern linear robust control, of which $H_{\infty }$ loop-shaping is one methodology, has been successfully used in diverse fields, such as control of combustion instabilities [18–20], control of developing flow [21], voltage converter design [22] and vehicle oscillation damper optimization [23], to achieve control even in nonlinear systems with limit cycles. $H_{\infty }$ loop-shaping combines classical loop-shaping, which can obtain tradeoffs between good performance and robust stability, with modern $H_{\infty }$ optimization in order to guarantee closed-loop stability and a level of robust stability at all frequencies [16,17]. It can be applied to plants with high dimensions and is computationally inexpensive [19], at least for plants of low and moderate order. The resulting controllers generally have orders comparable to those of the plants.

It is typical to approximate the OLTF as a high-order rational transfer function (RTF) for the purposes of controller design, meaning that high-order controllers will be generated. These are less reliable to implement than low-order controllers. As a final step, this work proposes a low-order controller design method, which combines $H_{\infty }$ loop-shaping, the *ν*-gap metric and a low-order fitting procedure.

The remainder of the paper is organized as follows. Section 2 presents the ducted laminar flame combustor model used as a test case in the paper, including the coupling of linear modelling for the acoustic waves with the nonlinear flame response. Section 3 demonstrates the determination of the nonlinear FDF from simulations using a level set approach (LSA), ensuring that our flame model is grounded in flow physics. Section 4 analyses the resulting nonlinear thermoacoustic instabilities for different flame positions. Section 5 presents the design of linear controllers which are guaranteed to be stabilizing from within limit cycle oscillations, using $H_{\infty }$ loop-shaping and the *ν*-gap metric. This includes the design of standard high-order controllers and a method for obtaining low-order controllers. The viscothermal damping of acoustic waves in ducts is presented in appendix, and conclusions are drawn in the final section.

## 2. Combustor model

A geometrically simple model combustor is considered for this study. The geometrical simplicity permits us to embed the advanced modelling features that capture the important phenomena at play in real combustors. This includes linear acoustic wave behaviour with realistic boundary and viscothermal losses, and complex nonlinear flame kinematics for sufficiently high amplitude perturbations.

**Figure 1.**
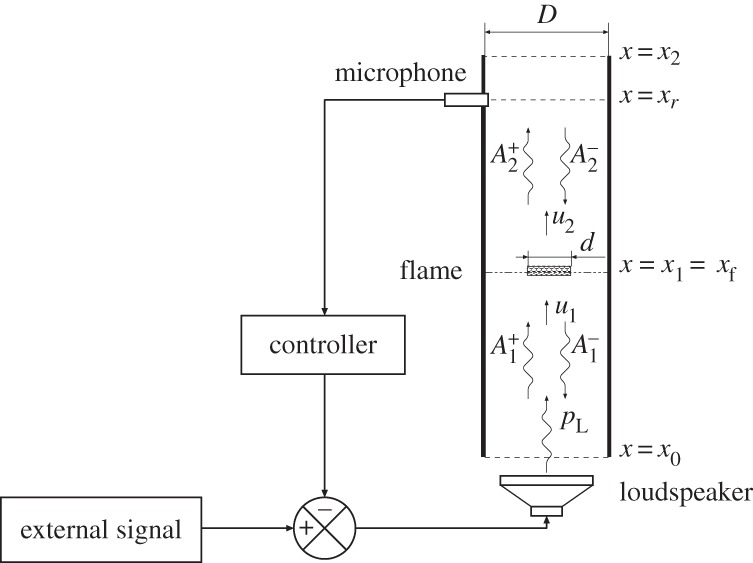
Schematic view of the Rijke tube and the feedback control configuration.

The model considered is a ducted laminar flame combustor, often known as a Rijke tube. It is shown schematically in figure 1, comprising a cylindrical Rijke tube with both ends open to the atmosphere. Denoting the distance along the duct axis by the vector *x*, the tube inlet and outlet are at *x*=*x*_0_=0 and *x*=*x*_2_=*l*, respectively. The inner diameter of the tube is *D*, and a laminar premixed methane–air conical flame is located at *x*=*x*_1_=*x*_*f*_. The Bunsen-type burner has an axisymmetric geometry with outlet diameter *d*. A loudspeaker is located at the bottom of the tube as an actuator for control, with a pressure sensor located a distance *x*=*x*_*r*_ downstream of the combustion zone. The modelling analysis makes the following assumptions: (i) the combustion zone is considered ‘compact’ compared with the acoustic wavelength, and we limit attention to low frequencies where we need to account only for longitudinal waves [24]. (ii) The fluids before and after the flame are assumed to be perfect gases [24]. (iii) There is no noise produced by the entropy waves formed during the unstable combustion process—these waves are assumed to leave the tube without interaction with the flow at the end of tube [25]. (iv) The flame remains stabilized at the burner outlet. Flame intrinsic instabilities [26] and irregular response to strong disturbances [27] are not accounted for.

The flow can be taken to be composed of a steady uniform mean flow (denoted $\bar{\;}$ ) and small perturbations (denoted ′). Harmonic time variations are considered for which all fluctuating variables have the form $a^{\prime }=\hat{a}\;e^{i\omega t}$, where *ω* is complex angular frequency. The regions upstream and downstream of the flame are indicated by the subscripts 1 and 2, respectively. Then, the pressure, *p*, and longitudinal velocity, *u*, in the two regions can be expressed in terms of the amplitudes $A_{n}^{+}$ and $A_{n}^{-}$ of the upward and downward propagating acoustic waves, respectively, their wavenumbers, $k^{\pm }={\bar{k}}^{\pm }+\Delta k^{\pm }$, where ${\bar{k}}^{\pm }=\omega /(\bar{c}\pm \bar{u})$ and Δ*k*^±^ are wavenumber corrections for the viscothermal damping effects (see appendix), and the speed of sound, *c*, and density, *ρ* [28]. The pressure, *p*, and longitudinal velocity, *u*, in the two regions are expressed as

2.1 $$\begin{array}{rl} p_{n}(x,t) & ={\bar{p}}_{n}+{\hat{p}}_{n}(x)\;e^{i\omega t}\\ & ={\bar{p}}_{n}+(A_{n}^{+}e^{ik_{n}^{+}(x-x_{n-1})}+A_{n}^{-}e^{-ik_{n}^{-}(x-x_{n-1})})\;e^{i\omega t}\;(n=1,2)\\ \;\text{and}\;\;u_{n}(x,t) & ={\bar{u}}_{n}+{\hat{u}}_{n}(x)\;e^{i\omega t}\\ & ={\bar{u}}_{n}+\frac{1}{{\bar{\rho }}_{n}{\bar{c}}_{n}}(A_{n}^{+}e^{ik_{n}^{+}(x-x_{n-1})}-A_{n}^{-}e^{-ik_{n}^{-}(x-x_{n-1})})\;e^{i\omega t}. \end{array}\}$$

The acoustic wave strengths, $A_{n}^{+}$ and $A_{n}^{-}$, are related by pressure reflection coefficients^1^ at the tube boundaries—the inlet and outlet pressure reflection coefficients are characterized by *R*_1_ and *R*_2_, respectively.

For an open duct, radiation of sound from the duct ends can be accounted for using frequency-dependent reflection coefficients that act as low-pass filters [29]. The tube also appears acoustically longer than its physical length, and this end correction can be accounted for. The reflection coefficient for an unflanged pipe end can be approximately expressed in the Laplace domain as [29,30]

2.2 $$R=-(1+2\tau_{d}^{2}s^{2})e^{-0.61\tau_{d}s}\;\text{for}\;2\pi f\tau_{d}\ll 1.$$

where $\tau_{d}=D/\bar{c}$. It was confirmed in [31] that the gain of this reflection coefficient decreases with both duct diameter and frequency. The reflection coefficients at the inlet and outlet of the Rijke tube are denoted by *R*_1_ and *R*_2_, respectively.

In order to relate the acoustic wave strengths across the flame, the linearized mass, momentum and energy conservation equations across the flame zone (*x*=*x*_f_) are combined with the perfect gas equation. Denoting the jump across the flame as ${\left[\cdot \right]}_{1}^{2}$, the amplitude of the flame’s heat release rate perturbation as $\hat{\dot{Q}}$ and the cross-sectional area of the duct as *S*, this yields two equations which are independent of the downstream density, *ρ*_2_, which contains an entropy component [32]

2.3 $$\frac{S_{2}}{S_{1}}{\left[\hat{p}(x_{f})\right]}_{1}^{2}+{\bar{\rho }}_{1}{\bar{u}}_{1}{\left[\hat{u}(x_{f})\right]}_{1}^{2}+{\left[\bar{u}\right]}_{1}^{2}\left({\bar{\rho }}_{1}{\hat{u}}_{1}(x_{f})+\frac{{\hat{p}}_{1}(x_{f})}{{\bar{c}}_{1}^{2}}{\bar{u}}_{1}\right)=0$$

and

2.4 $$\frac{\gamma }{\gamma -1}{\left[S(\bar{p}\hat{u}(x_{f})+\hat{p}(x_{f})\bar{u})\right]}_{1}^{2}+{\bar{\rho }}_{1}{\bar{u}}_{1}S_{1}{\left[\bar{u}\hat{u}(x_{f})\right]}_{1}^{2}=\hat{\dot{Q}}.$$

Note that upstream of the flame, *S*_1_=*π*(*D*^2^−*d*^2^)/4. *γ* is the ratio of specific heats of the gases, considered constant throughout the duct owing to the small temperature jump across the flame. Substituting the linearized pressure and velocity expressions from equation (2.1) together with the boundary conditions into the above two conservation equations, the governing equations linking the acoustic wave strengths before and after the flame are obtained in terms of *s*, the Laplace transform variable [33] (where $s_{n}=s+\Lambda_{n}\sqrt{s}$, *n*=1,2, the second term accounting for viscothermal damping with the coefficient *Λ*_*n*_ defined in equation (A 2)), time delays $\tau_{n}^{\pm }=(x_{n}-x_{n-1})/({\bar{c}}_{n}\pm {\bar{u}}_{n})$ and $\Xi =(S_{1}/S_{2})({\bar{\rho }}_{2}{\bar{c}}_{2})/({\bar{\rho }}_{1}{\bar{c}}_{1})$. The governing equation is expressed as

2.5 $$\underset{{\text{M}}_{\text{0}}}{\underbrace{\left[\begin{array}{cccc} 1 & -R_{1} & 0 & 0\\ M_{1}e^{-\tau_{1}^{+}s_{1}} & M_{2}e^{\tau_{1}^{-}s_{1}} & -M_{3} & -M_{4}\\ M_{5}e^{-\tau_{1}^{+}s_{1}} & -M_{6}e^{\tau_{1}^{-}s_{1}} & -\frac{1}{\Xi }M_{7} & \frac{1}{\Xi }M_{8}\\ 0 & 0 & -R_{2}e^{-\tau_{2}^{+}s_{2}} & e^{\tau_{2}^{-}s_{2}} \end{array}\right]}}\underset{A}{\underbrace{\left[\begin{array}{c} A_{1}^{+}\\ A_{1}^{-}\\ A_{2}^{+}\\ A_{2}^{-} \end{array}\right]}}=\left[\begin{array}{c} 0\\ 0\\ -\frac{\gamma -1}{{\bar{c}}_{1}S_{1}}\hat{\dot{Q}}\\ 0 \end{array}\right].$$

The matrix coefficients, $M_{n}$, are given in appendix. In order to close equation (2.5) that contains five unknowns ($A_{n}^{\pm }$ and $\hat{\dot{Q}}$), a flame model relating $\hat{\dot{Q}}$ to the acoustic fluctuations is needed. In this work, we use an FDF, whose form is derived numerically in §3.

Although the combustor configuration contains only two acoustic modules (the two regions upstream and downstream of the flame), the system is complex owing to the frequency-dependent pressure reflection coefficients, viscothermal damping terms and the presence of flame nonlinearity. A numerical solver is used to solve the nonlinear eigenproblem [34]. The solver is part of *OSCILOS*, an open source combustion instability low-order network simulation tool. It represents complicated combustor geometries as a network of simple connected modules, modelling the acoustic wave behaviour analytically using linear wave-based methods, and able to incorporate a wide range of linear and nonlinear flame models. It can predict thermoacoustic modal frequencies, growth rates, mode shapes and the time evolution of disturbances, and has been validated against experiments [35].

In this work, a relatively low cost numerical method, compared with large eddy simulations [35,36], is used to determine the FDF of the laminar conical premixed flame. This numerical method is described in §3.

## 3. Determining the flame describing function

In the model combustor, a laminar conical premixed flame within the duct generates a mean and fluctuating heat release rate. The heat release rate is taken to be directly proportional to the flame’s surface area, which means that an accurate flame model for insertion into equation (2.5) can be found by capturing the kinematic response of the flame to oncoming acoustic disturbances.

The instantaneous location of a moving flame front subjected to oncoming flow perturbations can be captured using the LSA proposed by Markstein [37]. This kinematic model is also known as the *G*-equation model, and treats the flame as an extremely thin interface (corresponding to the iscontour *G*=0), separating fresh reacting flow (denoted by *G*<0) from the burned gases (*G*>0). The flame front is assumed to propagate normal to itself with the velocity **U**⋅**n**−*s*_L_, where **U** is the fresh reacting flow velocity vector and in this work is two-dimensional, **U**=(*u*_*x*_,*u*_*r*_), owing to the axisymmetric configuration. *s*_L_ is the flame burning speed and **n**=**∇***G*/|**∇***G*| is the normal vector. The transport equation for *G* is [38]

3.1 $$\frac{\partial G}{\partial t}+\text{U}\cdot \nabla G=s_{L}|\nabla G|.$$

The mutual interactions of the flame front kinematics and the hydrodynamic flow field are not accounted for [39], meaning the fresh mixture flow field above the burner outlet and inside the flame can be treated using a simple model which is independent of the flame evolution. It has been shown that when the *G*-equation model is combined with a simple incompressible velocity perturbation model in the fresh mixture, complex flame front evolution phenomena, including the ‘pinch-off’ that occurs for strong flow perturbations, can be captured [40,41]. This model was recently validated using direct numerical simulations [42], and is expressed as

3.2 $$u_{x}={\bar{u}}_{1}+u_{1}^{\prime }={\bar{u}}_{1}+{\hat{u}}_{1}\cos(kx-\omega t)\;\mathrm{and}\;u_{r}=u_{r}^{\prime }=k\frac{r}{2}{\hat{u}}_{1}\sin(kx-\omega t).$$

The composition of the fresh reacting flow is considered constant, and the dependence of the flame burning speed on the flame front curvature is taken to be $s_{L}=s_{L0}(1-L\kappa )$, where *s*_L0_ is the burning speed of a laminar planar flame, L is the Markstein length, which depends on mixture equivalence ratio *ϕ* and thermal conditions [43] (and hence is constant in this work), whereas *κ*=**∇**⋅**n** is the local flame curvature [37,43].

The *G*-equation (equation (3.1)) combined with the incompressible flow velocity model (equation (3.2)) and the flame curvature effect is solved numerically using a fifth-order weighted essentially non-oscillatory (WENO) scheme [44] for spatial discretization and a third-order total variation diminishing (TVD) Runge–Kutta scheme for time integration [45]. The spatial and temporal resolutions are fixed in all calculations: Δ*x*=Δ*r*=5×10^−3^*d* and $\Delta t=5\times 10^{-4}d/{\bar{u}}_{1}$, respectively, where *d* is the diameter of the burner outlet. To significantly reduce the computational cost, a local LSA is employed, only accounting for the grids around the flame front [46]. The boundary condition for the centreline of the flame is symmetry—flame axisymmetry is exploited to simulate only half of the flame. The flame base is considered always attached to the burner lip, with *G* at the flame base reinitialized to 0 at every simulation time step. No boundary condition is needed for other boundaries, because the local LSA is implemented.

For premixed flames, the heat release rate can be calculated using

3.3 $$\dot{Q}(t)=2\pi {\bar{\rho }}_{1}s_{L0}{\dot{\omega }}_{T}\iint_{A}(1-L\kappa )|\nabla G|\delta (G)\;dr\;dx,$$

where ${\bar{\rho }}_{1}$ denotes the mean density of the fresh reacting flow, ${\dot{\omega }}_{T}$ indicates the heat release rate per volume from combustion and A indicates the whole space of the LSA simulation. *δ*(*G*) is the delta function, representing integration over the flame front, which is performed using a high-order scheme [47].

The weakly nonlinear flame model to be extracted from these simulations is in the form of an ‘FDF’, expressed as

3.4 $$F({\hat{u}}_{1}/{\bar{u}}_{1},s)=\frac{{\hat{\dot{Q}}}_{1}({\hat{u}}_{1}/{\bar{u}}_{1},s)/\bar{\dot{Q}}}{{\hat{u}}_{1}(s)/{\bar{u}}_{1}},$$

where ${\hat{\dot{Q}}}_{1}({\hat{u}}_{1}/{\bar{u}}_{1},s)$ is the Laplace transform of the fundamental term of heat release rate perturbations. Note that this can be thought of as an ‘input amplitude dependent’ transfer function or frequency response.

**Figure 2.**
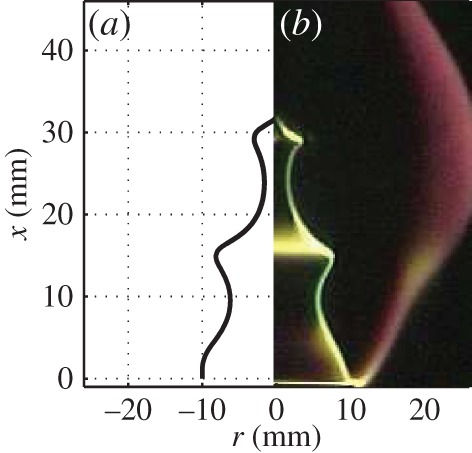
Comparisonbetween locations of the flame front deduced using LSA (*a*) and Schlieren techniques [48,49] (*b*) of a laminar conical premixed flame when the flame is modulated at *f*_p_=100 Hz for a perturbation level ${\hat{u}}_{1}/{\bar{u}}_{1}=0.1$. $t/T=\frac{1}{20}$, where $T=1/f_{p}$. In the Schlieren image, the yellow part represents the flame front, and the red part indicates the hot plume surrounding the flame. (Online version in colour.)

**Figure 3.**
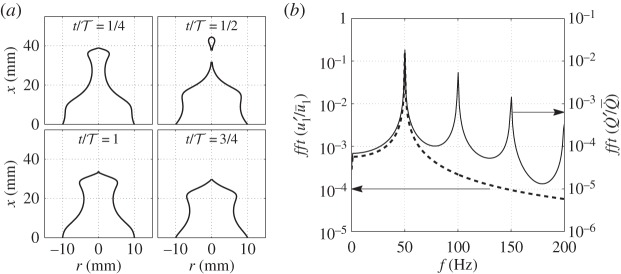
(*a*) Flame front at four instants in a forcing cycle. $t/T=\frac{1}{4},\frac{1}{2},\frac{3}{4}$ and 1. *f*_p_= 50 Hz. ${\hat{u}}_{1}/{\bar{u}}_{1}=0.15$. (*b*) Spectra of $u_{1}^{\prime }/{\bar{u}}_{1}$ (represented by the dashed line and corresponding to the left vertical axis) and ${\dot{Q}}^{\prime }/\bar{\dot{Q}}$ (represented by the continuous line and corresponding to the right vertical axis).

To validate the LSA, a flame for which experimental data are available is firstly simulated. A laminar conical methane–air premixed flame with burner outlet diameter *d*=20 mm, equivalence ratio *ϕ*=1.0, mean velocity ${\bar{u}}_{1}=1.5\;m\;s^{-1}$, laminar flame speed *s*_L0_=0.368 m s^−1^ and Markstein length L=1 mm [43] is considered. Figure 2 compares the simulated flame front location from the LSA with a four-colour Schlieren image from experiments, when the perturbation frequency and level are *f*_p_=100 Hz and ${\hat{u}}_{1}/{\bar{u}}_{1}=0.1,$ respectively. The flame front is seen to respond strongly even for a relatively weak perturbation level. The prediction matches the experimental result with the flame cusps accurately captured. The evolution of the flame front throughout an entire forcing cycle for modulation at *f*_p_=50 Hz and ${\hat{u}}_{1}/{\bar{u}}_{1}=0.15$ is shown in figure 3*a*. At $t/T=\frac{1}{2}$, where $T=1/f_{p}$, pinch-off occurs and a flame pocket is produced. The frequency spectrum of the heat release rate perturbations is shown for 50 Hz forcing in figure 3*b*. Although the nonlinear flame response causes harmonics at high frequencies, the fact that the dominant flame response frequency exactly matches the forcing frequency validates the ‘weakly’ nonlinear assumption of the FDF.

**Figure 4.**
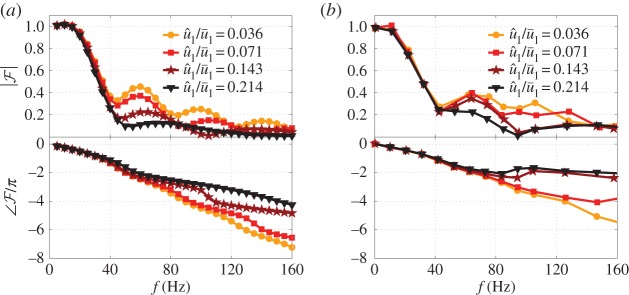
FDF of a laminar conical premixed methane–air flame deduced from the LSA (*a*) and experiments [50] (*b*). Top graph: gain. Bottom graph: phase. Mean flow velocity is ${\bar{u}}_{1}=2.12\;m\;s^{-1}$ and equivalence ratio is *ϕ*=1.08. Burner diameter is *d*=22 mm. (Online version in colour.)

The FDF, F, calculated using the LSA is compared with experimental results for a similar laminar conical premixed flame [50], with almost the same burner outlet diameter. The simulation and experimentally measured FDFs are shown in figure 4. The FDF has the shape of low-pass filter at constant ${\hat{u}}_{1}/{\bar{u}}_{1}$, with nonlinearity evident at higher frequencies—the gain of FDF decreases with increasing ${\hat{u}}_{1}/{\bar{u}}_{1}$, but with the response linear at low frequencies even for a large ${\hat{u}}_{1}/{\bar{u}}_{1}=0.214$. The phase dependence on forcing amplitude begins at a higher frequency than the gain dependence. The gain and phase lag of the FDF both generally decrease with modulation level, which is consistent with the experimental results. It can clearly be concluded that the LSA captures both the flame front motion and the main features of the FDF of laminar conical premixed flames.

**Figure 5.**
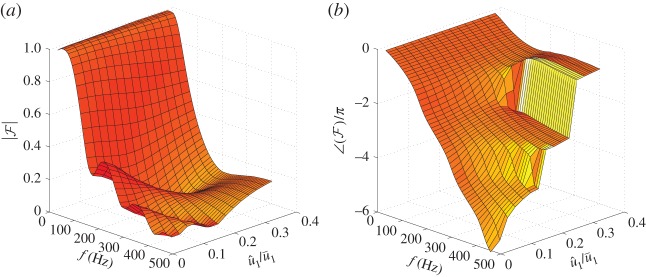
The gain (*a*) and phase (*b*) of the FDF determined by the LSA for a laminar conical premixed methane–air flame. ${\bar{u}}_{1}=1.5\;m\;s^{-1}$. *d*=6 mm. *ϕ*=1.0. (Online version in colour.)

Simulation is then performed for the combustor test-case flame. For a laminar conical premixed flame, the cut-off frequency of the FDF is inversely proportional to the diameter of the burner outlet [6,51,52]. To guarantee that a sufficiently large heat release feeds the unstable thermoacoustic mode, the diameter of the burner outlet is reduced to *d*=6 mm (meaning that the cut-off frequency is approximately three times that of the FDF shown in figure 4), with the other flow parameters unchanged. Simulations are performed for modulation frequencies ranging from 10 to 500 Hz in intervals of 10 Hz, and with normalized velocity perturbation levels ranging from 0.025 to 0.4 in intervals of 0.025. The FDF is shown in figure 5. The shape remains similar, with the response generally falling off with both frequency and modulation level. Although |F| does not strictly decrease with increasing ${\hat{u}}_{1}/{\bar{u}}_{1}$ above 300 Hz, the accuracy of the flow velocity perturbation model (equation (3.2)) breaks down at high frequencies. With the dominant unstable frequency in the Rijke tube being 210 Hz, the FDF results can be considered accurate below 300 Hz. The FDF provided by the LSA simulations (denoted $F_{\mathrm{LSA}}$) is discrete in both frequency and forcing amplitude and is limited to the frequency range [10, 500] Hz. The FDF can be considered a set of flame transfer functions (FTFs)^2^ for different ${\hat{u}}_{1}/{\bar{u}}_{1}$, each of which depends only on *s*. A fitting procedure is performed on each FTF using the Matlab command *fitfrd*^3^ . The resulting state-space models for different ${\hat{u}}_{1}/{\bar{u}}_{1}$, each of order 14, are combined to give a ‘fitted FDF’, denoted F. The maximum error for each fitting procedure is smaller than 0.005, for *f*∈[10,500] Hz and ${\hat{u}}_{1}/{\bar{u}}_{1}\in [0.025,0.4]$.

## 4. Simulation and analysis of the unstable combustor

By inserting the fitted FDF F into the acoustic wave equation (2.5), the thermoacoustic stability of the combustor is characterized by a matrix equation involving the acoustic matrix, **M**_**0**_, from equation (2.5), a matrix, **M**_**1**_, containing the FDF and the acoustic wave strength vector, A, from equation (2.5)

4.1 $$({\text{M}}_{\text{0}}+{\text{M}}_{\text{1}})A=\text{0},\;\text{where}\;{\text{M}}_{\text{1}}=QF\left({\hat{u}}_{1}/{\bar{u}}_{1},s\right)=\left[\begin{array}{cccc} 0 & 0 & 0 & 0\\ 0 & 0 & 0 & 0\\ e^{-\tau_{1}^{+}s_{1}} & -e^{\tau_{1}^{-}s_{1}} & 0 & 0\\ 0 & 0 & 0 & 0 \end{array}\right]$$

where $Q={\bar{T}}_{2}/{\bar{T}}_{1}-1$ and $\bar{T}$ denotes the mean flow temperature. The thermoacoustic modes are given by the values of *s*=i*ω*=*σ*+i*ω*_i_ at constant ${\hat{u}}_{1}/{\bar{u}}_{1}$ for which $\det({\text{M}}_{\text{0}}+{\text{M}}_{\text{1}})=0$, where det denotes the matrix determinant, *ω*_i_=2*πf* is real angular frequency and *σ* is the growth rate. The stability of the mode is defined by the sign of the growth rate, with a positive value corresponding to an unstable mode. This calculation is performed within *OSCILOS*. The combustor parameters considered for the analysis are listed in table 1.

**Table 1.** Parameters used in the analysis. They are fixed, unless otherwise stated.

*l* [m]	1
*D* [mm]	30
*d* [mm]	6
*x*_*r*_ [m]	0.5
*γ* [–]	1.4
${\bar{p}}_{1}$ [Pa]	101325
${\bar{\rho }}_{1}$ [kg m^−3^]	1.18
${\bar{u}}_{1}$ [m s^−1^]	1.5
${\bar{T}}_{1}$ [K]	300
${\bar{T}}_{2}$ [K]	600
${\bar{c}}_{1}$ [m s^−1^]	347
${\bar{c}}_{2}$ [m s^−1^]	491
*ϕ*	1.0
*s*_L0_ [m s^−1^]	0.368
L [mm]	1

**Figure 6.**
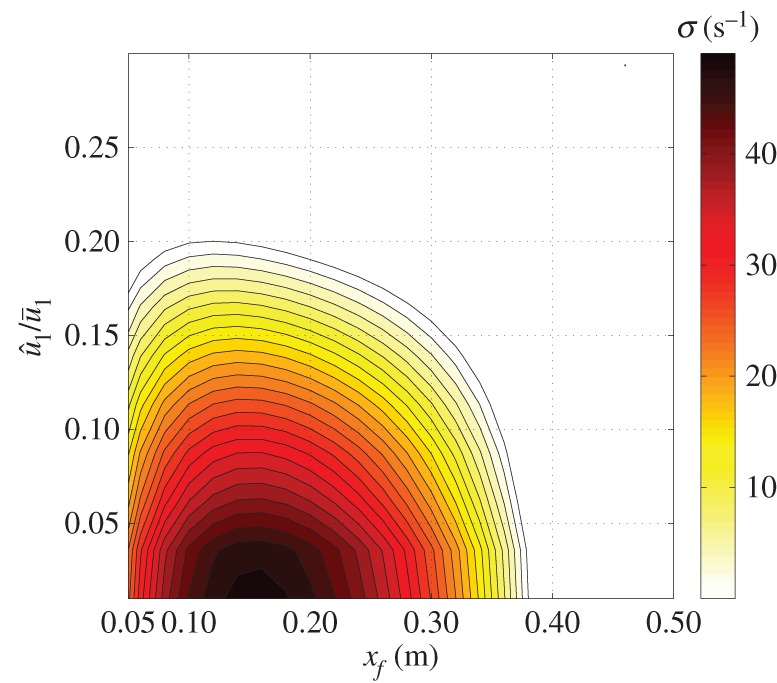
Contour plot of the positive growth rate *σ* of the unstable mode with *x*_f_ and ${\hat{u}}_{1}/{\bar{u}}_{1}$. (Online version in colour.)

**Figure 7.**
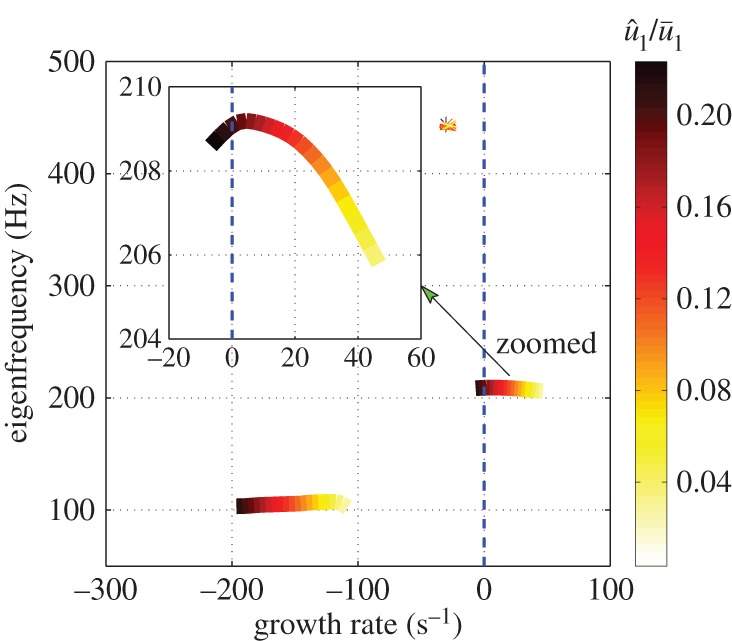
Trajectories of eigenvalues with increasing ${\hat{u}}_{1}/{\bar{u}}_{1}$. *x*_f_=0.2 m. The mode with frequency ∼100 Hz is introduced by the time delay of the FDF and is always stable. (Online version in colour.)

It is found that only the mode with the eigenfrequency *f*≈210 Hz has the potential for instability (this corresponds to the cold flow fundamental frequency of 173 Hz for perfect acoustic reflections from the boundaries). This is consistent with both the FDF gain fall-off and the acoustic losses increasing with frequency, having a stabilizing effect on higher-order modes. A growth rate map for this unstable mode as a function of both flame position within the duct and flame velocity perturbation level is shown in figure 6, with only positive (unstable) growth rates shown. The most dangerous unstable position is a quarter of the way along the duct, which is generally the situation in a Rijke tube [53]. For each flame location, limit cycle oscillations will be established at the velocity fluctuation level corresponding to the uppermost contour, as this is where the growth rate has dropped to zero. The flame location, *x*_f_=0.2 m, is chosen for feedback controller design, corresponding to the limit cycle being established at a relatively large ${\hat{u}}_{1}/{\bar{u}}_{1}$. Figure 7 shows the trajectories of the growth rate and eigenfrequency with increasing ${\hat{u}}_{1}/{\bar{u}}_{1}$ for this flame location. For weak perturbations, the growth rate is strongly positive, implying a rapidly growing exponential envelope, e.g. for ${\hat{u}}_{1}/{\bar{u}}_{1}=0.025$, the growth rate is 46 s^−1^, implying exponentially growth with an envelope of exp(46t). This growth rate decreases with ${\hat{u}}_{1}/{\bar{u}}_{1}$, becoming zero when ${\hat{u}}_{1}/{\bar{u}}_{1}=0.18$, hence this is the velocity fluctuation amplitude at which we expect limit cycle oscillations to occur.

## 5. Feedback control from within limit cycle oscillations

### (a) Open-loop transfer function

**Figure 8.**
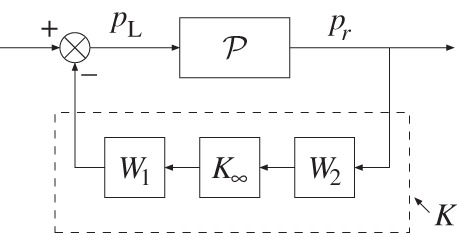
The sketch of the negative feedback control system.

Actuators for feedback control of combustion instabilities generally fall within two categories. The first seeks to modify the fuel–air composition, normally via a fuel valve, whereas the second uses acoustic actuators to modify the pressure waves inside the combustor directly [1,18]. This work assumes the latter—these are generally better suited to light duty combustors. A loudspeaker at the bottom end of the duct is assumed, injecting a pressure signal *p*_L_. A microphone mounted *x*_*r*_ = 0.5 m downstream of the flame measures the pressure at that location. To simplify the analysis, we do not account for the instrument transfer functions of the loudspeaker and microphone [13,54]: we consider that the electrical driving signal to the loudspeaker *I*_in_ equals *p*_L_, and the microphone electrical output signal *I*_out_ equals the measured pressure signal *p*_*r*_. Furthermore, we assume that the fuel–air ratio is unaffected by the acoustic waves, maintaining the applicability of the FDF calculated in §3. For model-based feedback controller design (figure 8), it is necessary to have a model for the OLTF from *p*_L_ to *p*_*r*_:

5.1 $$P(s)=\frac{p_{r}(s)}{p_{L}(s)}=B{\left({\text{M}}_{\text{0}}+{\text{M}}_{\text{1}}\right)}^{-1}C,$$

where

5.2 $$B=[0,0,e^{-\tau_{r}^{+}s_{2}},e^{\tau_{r}^{-}s_{2}}]\;\text{and}\;C={\left[1,0,0,0\right]}^{T}$$

and the time delays corresponding to the microphone position are $\tau_{r}^{\pm }=(x_{r}-x_{f})/({\bar{c}}_{2}\pm {\bar{u}}_{2})$.

**Figure 9.**
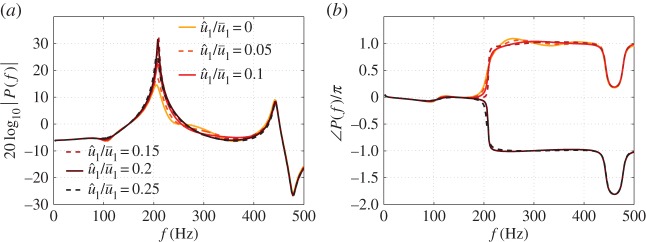
Evolutions of the gain (*a*) and phase (*b*) of the OLTF *P* with *f* for different ${\hat{u}}_{1}/{\bar{u}}_{1}$. (Online version in colour.)

Owing to the flame nonlinearity, P(s) is nonlinear and depends on ${\hat{u}}_{1}/{\bar{u}}_{1}$. Following weakly nonlinear theory, it can be cast into a set of linear OLTFs, P(s)∈P(s), with a different *P*(*s*) for each value of ${\hat{u}}_{1}/{\bar{u}}_{1}$. The OLFTs for different ${\hat{u}}_{1}/{\bar{u}}_{1}$ when *x*_f_=0.2 m are shown in figure 9.

Each modal peak is associated with a phase change of *π*, implying a second-order transfer function contribution. The direction of the phase change provides stability information—a phase increase in *π* corresponds to an unstable conjugate pair of poles, whereas a phase decrease in *π* indicates a stable conjugate pair. From figure 9, normalized velocity perturbation amplitudes, ${\hat{u}}_{1}/{\bar{u}}_{1}$, of 0–0.15 correspond to unstable OLTFs, whereas amplitudes of 0.2 and 0.25 correspond to stable OLTFs. This is consistent with the previous finding that limit cycle oscillations occur at ${\hat{u}}_{1}/{\bar{u}}_{1}=0.18$.

It is thus clear that a controller design based on just one of these linear OLTFs cannot guarantee the entire set of plants. In order to guarantee stability from within limit cycle oscillations, a robust controller must

— reduce the size of perturbations when the system is in limit cycle, with the effect of reducing ${\hat{u}}_{1}/{\bar{u}}_{1}$. The set of plants in this regime appear open loop stable and
— result in closed loop stability for all plants. This requires a sufficiently large stability margin.

### (b) $H_{\infty }$ loop-shaping and the *ν*-gap metric

$H_{\infty }$ loop-shaping is used to design a robust stabilizing controller. $H_{\infty }$ loop-shaping combines classical loop-shaping design with guaranteed robustness at all frequencies. It involves designing a pre- or post-compensator to ‘shape’ the singular values (i.e. the gain for single-input–single-output (SISO) systems) of the open-loop system, so that they have a high gain where disturbance rejection is important, and a low gain where robustness to multiplicative plant uncertainty is required (see references [17,55] for an explanation of different types of uncertainty from a control systems perspective). A stabilizing controller is then synthesized using Matlab (e.g. using the *ncfsyn* command), which minimizes the $H_{\infty }$ norm of the closed-loop transfer function matrix (see equation (5.5))—this norm also provides a measure of the system robustness. If it is suitably small, then a robust stabilizing controller has been found. The gain of the shaped transfer function will then not be significantly affected by the controller, but the phase will be altered so as to achieve stability [17]. If the norm is not suitably small then another iteration on the choice of the pre- or post-compensator must be performed. The underpinning mathematics can be found in [17].

The shape of the open-loop plant *P* is first modified using pre- and post-compensators *W*_1_ and *W*_2_ (figure 8). For an SISO system, modifying either one of these has the same effect. The $H_{\infty }$ controller $K_{\infty }$ is then synthesized in Matlab, with the final feedback controller *K*, being $K=W_{1}K_{\infty }W_{2}$.

The *ν*-gap metric provides a useful measure of the distance in the state space between two open-loop plants with respect to how they behave when connected in a unity feedback control loop [16]. For SISO systems, it can be computed from the frequency response: the *ν*-gap, *δ*_*ν*_, between plants *P*_1_ and *P*_2_ is given by

5.3 $$\delta_{\nu }(P_{1},P_{2})=\underset{\omega }{\sup}\psi (P_{1}(i\omega ),P_{2}(i\omega )),\;{\mathrm{provided}\;\mathrm{the}\;\mathrm{winding}\;\mathrm{number}\;\mathrm{condition}\;\mathrm{is}\;\mathrm{satisfied}}^{4},$$

where^4^

5.4 $$\psi (P_{1}(i\omega ),P_{2}(i\omega ))=\frac{|P_{1}(i\omega )-P_{2}(i\omega )|}{{\left(1+|P_{1}(i\omega ){|}^{2}\right)}^{1/2}{\left(1+|P_{2}(i\omega ){|}^{2}\right)}^{1/2}}.$$

The *ν*-gap metric fits very naturally into $H_{\infty }$ loop-shaping control design, which returns both a feedback controller design, and the corresponding ‘stability margin’, $b_{\infty }$, for that controller/plant pairing. The stability margin $b_{\infty }$ for a shaped plant *P*_*W*_=*W*_1_*P*_1_*W*_2_ and the resulted controller $K_{\infty }$ is represented as [17]

5.5 $$b_{\infty }={∥\left[\begin{array}{c} I\\ P_{W} \end{array}\right]{\left(I-K_{\infty }P_{W}\right)}^{-1}[I\;K_{\infty }]∥}_{\infty }^{-1},$$

where $∥\cdot {∥}_{\infty }$ is the infinity norm. If the controller is applied to a different plant (e.g. *P*_2_), separated from the initial by *ν*-gap *δ*_*ν*_, as long as $b_{\infty }>\delta_{\nu }$, then the $H_{\infty }$ loop-shaping controller is also guaranteed to stabilize this second system.

### (c) $H_{\infty }$ loop-shaping controller design

**Figure 10.**
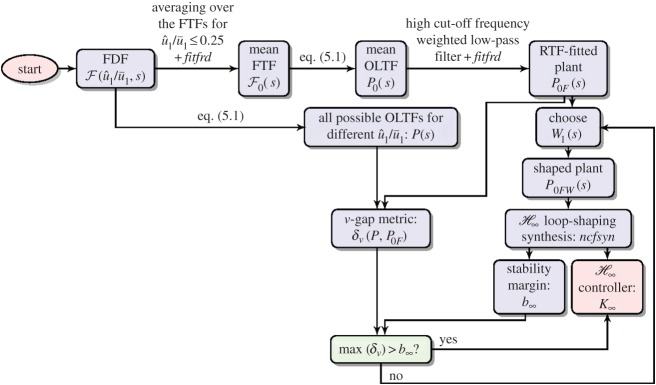
Flowchart of the $H_{\infty }$ loop-shaping controller design. Note that the post-compensator *W*_2_=*I*. (Online version in colour.)

$H_{\infty }$ loop-shaping controller design will need to be designed based on a nominal linear plant. The differences between this nominal plant and all of the possible OLTFs will then be characterized using the *ν*-gap metric. The controller design can be schematically explained by the flowchart shown in figure 10. For combustion instabilities, nonlinearities are introduced by the FDF, which can be treated as a discrete set of FTFs for different velocity perturbation levels ${\hat{u}}_{1}/{\bar{u}}_{1}$. From figure 6, limit cycle oscillations are generally established for ${\hat{u}}_{1}/{\bar{u}}_{1}\leq 0.2$. We thus calculate the mean FTF, $F_{0}$, by averaging over the FTFs for velocity perturbation levels ${\hat{u}}_{1}/{\bar{u}}_{1}\leq 0.25$. The mean FTF is fitted to a state space form in order to construct the mean OLTF (indicated by *P*_0_) for feedback controller design.

**Figure 11.**
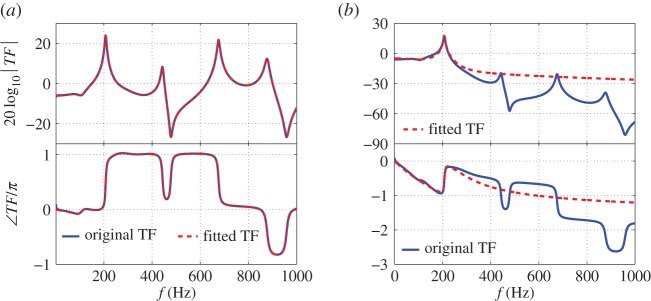
Comparisons between the original plant and fitted plant. (*a*) High-order fitting of *P*_0_. (*b*) Low-order fitting of *P*_0*W*_=*P*_0_*W*_1_. $W_{1}=W_{L}^{2}$ (see equation (5.7)). (Online version in colour.)

The presence of time delays in the wave-based models (e.g. see equations (4.1), and (5.2)) means the system is infinite dimensional. In order to implement $H_{\infty }$ synthesis, the mean OLTF needs to be approximated by a RTF which will be finite dimensional. The exponential terms could be replaced by Padé approximants [56], but the presence of complicated damping mechanisms means that this is not straightforward. In this work, a frequency-domain fitting procedure using the Matlab command *fitfrd* is applied to obtain a RTF approximation to the mean OLTF. As the FDF is only valid over the [10, 500] Hz frequency range, we fit the OLTF over a restricted frequency range [0, 1000] Hz, and also add a weighted low-pass filter to the RTF to reduce its gain at high frequencies. The resulting RTF, represented by *P*_0*F*_, typically has an order of 16. A comparison of the original infinite-dimensional and RTF-fitted plants is shown in figure 11*a*.

**Figure 12.**
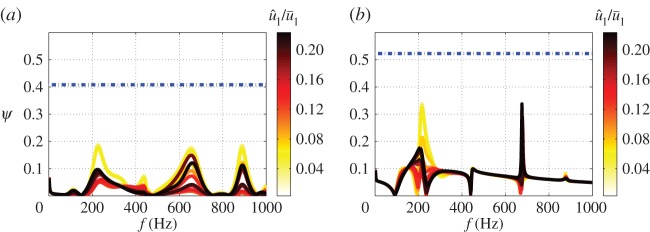
Plotof *ψ* with *f* for different ${\hat{u}}_{1}/{\bar{u}}_{1}$. (*a*) High-order fitting. *ψ*(*P*_0*F*_(i*ω*),*P*(i*ω*)). (*b*) Low-order fitting. *ψ*(*P*_0*WF*_(i*ω*),*P*_*W*_(i*ω*)). The dashed line represents the stability margin of the controller: $b_{\infty }$. (Online version in colour.)

As *P*_0*F*_ is SISO, we let the post-compensator *W*_2_=*I* and design the pre-compensator *W*_1_. A first-order low-pass filter with a cut-off frequency 300 Hz is chosen as *W*_1_, to drop the gain at high frequencies to provide robustness to multiplicative plant uncertainty. The *ν*-gaps between the plant *P*_0*F*_ and the plants *P* for different ${\hat{u}}_{1}/{\bar{u}}_{1}$ can be identified from the frequency variations of *ψ* (see equation (5.4)), shown in figure 12*a*. Large bumps occur at each mode, with the deviation reaching a maximum at the unstable frequency—210 Hz. The maximum value of the *ν*-gap across all different ${\hat{u}}_{1}/{\bar{u}}_{1}$ levels is $\max(\delta_{\nu })=0.18$.

A $H_{\infty }$ loop-shaping synthesis is then performed, using the Matlab command *ncfsyn* in the Robust control toolbox. The designed controller has an order of 16, and its stability margin is $b_{\infty }=0.41$, which is significantly larger than $\max(\delta_{\nu })$ above. This suggests that the controller should guarantee closed loop stability of all possible plants.

**Figure 13.**
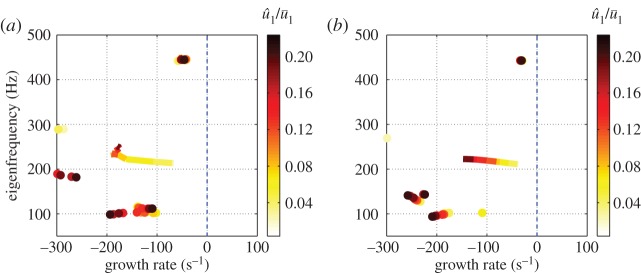
Trajectories of eigenvalues with increasing ${\hat{u}}_{1}/{\bar{u}}_{1}$ for the feedback control system. (*a*) High-order controller deign. (*b*) Low-order controller design. (Online version in colour.)

The performance of the negative feedback controller can be assessed by calculating the poles of the closed-loop transfer function P(s)/(1+K(s)P(s)), where P(s) is the original OLTF (equation (5.1)). Figure 13*a* shows the poles locations for different ${\hat{u}}_{1}/{\bar{u}}_{1}$ in the presence of the feedback controller. The growth rates of all modes are now stable, confirming closed loop stability for all ${\hat{u}}_{1}/{\bar{u}}_{1}$.

Of course, for sufficiently large values of ${\hat{u}}_{1}/{\bar{u}}_{1}$, the combustor exhibits limit cycle oscillations and the linear OLTF appears stable even without feedback control. For these conditions, the sensitivity transfer function provides an essential measure of the controller performance.

5.6 $$S(s)=\frac{1}{1+K(s)P(s)}.$$

**Figure 14.**
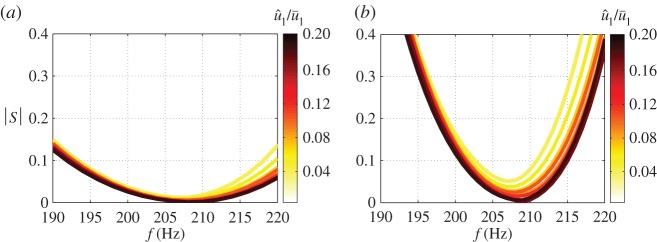
Evolution of |S| with frequency for different ${\hat{u}}_{1}/{\bar{u}}_{1}$. (*a*) High-order controller design. (*b*) Low-order controller design. (Online version in colour.)

When |*S*(i2*πf*)|<1, the feedback system attenuates disturbances compared with the open loop system, which corresponds to the amplitude of limit cycle oscillations and hence ${\hat{u}}_{1}/{\bar{u}}_{1}$ being reduced. Thus, assuming that the limit cycle frequency matches or is close to the frequency of the unstable mode, *f*^⋆^, the requirement now becomes |*S*(i2*πf*^⋆^)|<1^5^ . Figure 14*a* shows the plots of |*S*| versus frequency for different ${\hat{u}}_{1}/{\bar{u}}_{1}$. The unstable frequency is from 205 to 215 Hz in the amplitude range $0\leq {\hat{u}}_{1}/{\bar{u}}_{1}\leq 0.2$. The fact that |*S*(i2*πf*^⋆^)|<1 at these frequencies means that the current controller will reduce the limit cycle oscillation amplitude. For sufficient attenuation, the plant will appear unstable (see the yellow and orange colour lines in figure 9) and the feedback controller will then stabilize the system.

### (d) Low-order $H_{\infty }$ loop-shaping controller design

It has been shown that $H_{\infty }$ loop-shaping combined with the *ν*-gap metric can be used to design a robust controller that guarantees stabilization from within the limit cycle. The designed controller typically has an order comparable to that of the shaped plant [55]. High-order controllers are difficult to implement and exhibit lower reliability than low-order controllers. Although controller order reduction methods, such as balanced truncation methods [57], can be used on the plant or the controller, this work proposes an alternative approach to directly reduce the order of plant, provided that the maximum *ν*-gap $\left(\max(\delta_{\nu })\right)$ is smaller than the stability margin $b_{\infty }$.

The low-order method swaps the order of the shaping and fitting procedures. The plant *P*_0_ is firstly shaped with *W*_1_ using an ‘aggressive’ low-pass filter, which does not significantly alter the gain at low frequencies near the unstable mode, but reduces the gain at frequencies above the unstable mode, to provide robustness to fitting errors at these frequencies. A low-order fitting procedure is then implemented on the shaped plant *P*_0*W*_, with the fit over low frequencies near the unstable mode prioritized. A fourth-order low-pass filter is chosen as the pre-compensator *W*_1_ to shape the original plant, given by $W_{1}=W_{L}^{2}$, where

5.7 $$W_{L}(s)=\frac{\omega_{L}^{2}}{s^{2}+2\xi_{L}\omega_{L}s+\omega_{L}^{2}},$$

where *ω*_L_=2*π* × 200 s^−1^ and $\xi_{L}=\sqrt{2}/2$. *W*_1_ has a gain of 0.5 at 200 Hz, and so does not significantly alter the gain at the unstable frequency. The gain rapidly falls off to 0.025 at 500 Hz.

The controller designed by $H_{\infty }$ loop-shaping synthesis generally has a dimension comparable to that of the shaped plant, and so a fitted plant with low dimension is desirable. The shaped system contains only one unstable mode and exhibits fast gain fall-off at higher frequencies. The fitting procedure is performed on the shaped plant *P*_0*W*_=*P*_0_*W*_1_—this allows the dimension of the fitted RTF to be significantly reduced and yields a RTF with an order of 4. Figure 11*b* compares the original, *P*_0*W*_, and fitted, *P*_0*WF*_, plants. The fitted RTF captures the shape of original plant at low frequencies and has the shape of low-pass filter at high frequencies. Figure 12*b* shows the evolution of *ψ*(*P*_0*WF*_(i*ω*),*P*_*W*_(i*ω*)) with frequency. Compared with the previous approach (figure 12*a*), there are larger deviations at high frequencies ( *f*≥250 Hz) where the low-order fitting is less accurate. However, owing to the shape of *W*_1_, relatively small values of *ψ*(*P*_0*WF*_(i*ω*),*P*_*W*_(i*ω*)) are guaranteed for all frequencies and the maximum of *ν*-gap for all ${\hat{u}}_{1}/{\bar{u}}_{1}$ is $\max(\delta_{\nu })=0.34$.

A controller with an order of 3 is then obtained from the $H_{\infty }$ loop-shaping synthesis. The final controller $K=W_{1}K_{\infty }$ has an order of 7 in the denominator and 3 in the numerator of its RTF, which is straightforward to implement. The stability margin is $b_{\infty }=0.52$, which is larger than $\max\left(\delta_{\nu }\right)$.

The locations of the closed-loop poles for different ${\hat{u}}_{1}/{\bar{u}}_{1}$ are shown in figure 13*b*. The unstable mode is stabilized for all ${\hat{u}}_{1}/{\bar{u}}_{1}$, and the modes at high frequencies are not disturbed. However, the growth rate reduction for the unstable mode is less than for the high-order controller (figure 13*a*). This is due to the larger *ν*-gap caused by the low-order fitting. The gain of the sensitivity function *S*(*s*) with frequency is shown in figure 14*b* and is seen to be smaller than unity near the unstable mode frequency. This, combined with the fact that $b_{\infty }>\max\left(\delta_{\nu }\right)$, means that the low-order controller is also guaranteed to be stabilizing from within limit cycle oscillations.

**Figure 15.**
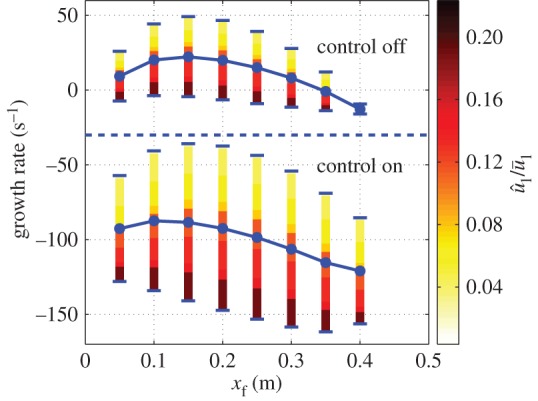
Plotof the growth rate of the unstable mode (*f*≈210 Hz) with and without control for different flame positions *x*_f_ and different velocity perturbation levels ${\hat{u}}_{1}/{\bar{u}}_{1}$. The dot markers represent the average growth rates. (Online version in colour.)

The robustness of the low-order controller design to changes in operating condition is investigated by moving the flame position along the combustor. Figure 15 shows that the growth rate of the dominant mode becomes negative for all unstable configurations. Thus, the controller designed stabilizes all possible flame locations within this combustor.

**Figure 16.**
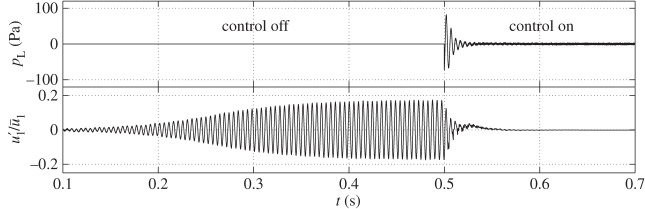
Evolution of *p*_L_ and $u_{1}^{\prime }/{\bar{u}}_{1}$ with time. The controller is switched on when *t*=0.5 s. *x*_f_=0.2 m.

The controller implementation in time domain simulations for the design condition of *x*_f_=0.2 m is shown in figure 16 (more information on the time domain simulation method is found in [31,34,58]). The normalized velocity perturbations before the flame, $u_{1}^{\prime }/{\bar{u}}_{1}$, and the actuation signal from the loudspeaker *p*_L_ are shown both before feedback control is activated and after it has been switched on. Prior to control, small disturbances are seen to grow rapidly, with a limit cycle established approximately beyond *t*=0.4 s. When the controller is switched on at *t*=0.5 s, the velocity perturbations decrease rapidly to the weak background white noise. The loudspeaker signal, *p*_L_, is initially large in order to attain control and decreases to a much smaller value in order to maintain control.

## 6. Conclusion

This article has presented a method of designing linear feedback controllers that are guaranteed to stabilize combustion instability, whenever they are activated. The guarantees are subject to some very reasonable assumptions on the nature of the flame nonlinearity that would be satisfied very widely in practice. A single feedback controller design then can guarantee stability whether control is activated within the regime of small linear disturbances and exponential growth or within the regime of limit cycle oscillations in which the amplitude has saturated.

The method combines the FDF approach for weakly nonlinear flame responses with $H_{\infty }$ loop-shaping for controller design and the *ν*-gap metric for characterizing nonlinearity as deviations from the ‘average’ linear model. The FDF gives rise to a ‘family’ of linear OLTFs corresponding to different flame perturbation levels. The maximum *ν*-gap between these and the average or nominal plant transfer function sets the minimum robustness stability margin required of the controller. An $H_{\infty }$ loop-shaping controller is then designed which has a stability margin exceeding this, and which also attenuates fluctuations within limit cycle.

The control strategy has been demonstrated on a representative model of a nonlinear combustion system comprising an unsteady laminar flame inside a Rijke tube. Linear plane waves within the duct are assumed to suffer damping owing to end radiation and viscothermal wall damping mechanisms. The nonlinear kinematic response of the flame to flow disturbances is modelled using an LSA, which combines the *G*-equation kinematic flame model with a two-dimensional flow velocity perturbation model and a flame front curvature-dependent burning speed. Simulations implementing this LSA have been validated by comparison with experimental data, and used to determine the FDF of the laminar conical premixed flame.

The calculated FDF of the target flame has been successfully incorporated into a recently developed code, *OSCILOS*, to calculate the eigenvalue evolutions of the nonlinear system with increasing flame velocity perturbation levels. The corresponding ‘family’ of OLTF between the monitored pressure perturbations downstream of the flame and the external pressure perturbations from an actuator at the entrance of the Rijke tube, has been established for the purpose of control.

A linear mean OLTF has been selected as the target plant for the controller design by averaging the FDF over a large range of velocity perturbation levels. The *ν*-gap metric has been applied to quantify the differences between the set of OLTFs for different perturbation levels and the selected plant. This metric fits naturally into the $H_{\infty }$ loop-shaping framework to provide the minimum required stability margin of the designed robust controller. An initial $H_{\infty }$ loop-shaping controller has been designed and shown to give effective robust control performance. Subsequently, a low-order $H_{\infty }$ loop-shaping controller based on an aggressively low-pass filtered weighted plant has been designed. Both controllers are guaranteed to stabilize the combustor for all possible nonlinear flame responses, as long as the *ν*-gap is smaller than the controller stability margin of the controller designed by $H_{\infty }$ loop-shaping synthesis. The resulting controller has been applied to simulations of the unsteady combustion system in the time domain and has been indeed found to suppress the combustion instability from within the limit cycle.

## Footnotes

1 The pressure reflection coefficient is defined as the ratio of reflected to incident acoustic waves at the boundary [24].

2 For small enough flow disturbances, the flame’s unsteady heat release rate generally responses linearly and can be described by a flame transfer function $F(s)=\hat{\dot{Q}}/\bar{\dot{Q}}/({\hat{u}}_{1}/{\bar{u}}_{1})$[40].

3 *fitfrd* returns a RTF of the best fit of a set of frequency-response data.

4 The winding number condition is: wno$(1+{\tilde{P}}_{2}(i\omega )P_{1}(i\omega ))+\eta (P_{1}(i\omega ))-\eta (P_{2}(i\omega ))=0$, where wno is the winding number, $\tilde{P}$ is the complex conjugate transpose of *P* and *η*(*P*) represents the number of open right-hand-plane poles of *P*[16].

5 It should be noted that the condition |*S*(i2*πf*^⋆^)|<1 is the sensitivity at the specific frequency, *f*^⋆^, that is relevant to reducing amplitude. It cannot be used to determine whether the plant over all frequencies is stabilized by the controller.

## Data accessibility

This paper has no data.

## Appendix A

**(a) Viscothermal damping of acoustic waves in a duct**

The propagation of sound waves in a duct has been found to be dissipated owing to viscothermal losses at the duct walls [30,59]. The forward and backward wavenumbers, ${\bar{k}}^{\pm }=-is/\bar{c}(1\pm \bar{M})$, are modified to $k^{\pm }={\bar{k}}^{\pm }+\Delta k^{\pm }$, where the correction approximately equals

A 1 $$\Delta k^{\pm }=\frac{-is^{1/2}}{\bar{c}(1\pm \bar{M})}\Lambda$$

with the coefficient *Λ* expressed as

A 2 $$\Lambda =\frac{2\nu^{1/2}}{D}\left(1+\frac{\gamma -1}{P_{r}^{1/2}}\right)$$

where *ν* and *P*_*r*_ are the kinematic viscosity and Prandtl number of gases, respectively. *D* is the diameter of the duct. *γ* is the ratio of specific heats. In the calculation, *P*_*r*_=0.71 is considered constant, both for air and combustion products of hydrocarbon flames. *ν* can be calculated by $\nu =\mu /\bar{\rho }$, with the dynamic viscosity *μ*_air_ of air calculated by the Sutherland’s Law [60], with further corrections expressed as

$$\mu_{\mathrm{air}}=\left\{ \begin{array}{ll} \mu_{\mathrm{ref}}{\left(\frac{T}{T_{\mathrm{ref}}}\right)}^{3/2}\frac{T_{\mathrm{ref}}+C_{S}}{T+C_{S}} & \mathrm{for}\;T\in [100,1000]\;K\\ a_{1}T+a_{0} & \mathrm{for}\;T\in [1000,3000]\;K, \end{array}\right.$$

where *μ*_ref_=1.716×10^−5^ kg m^−1^ s^−1^, *T*_ref_=273.15 K, *C*_*S*_=110.4 K, *a*_1_=2.653×10^−8^ and *a*_0_=1.573×10^−5^. It was found that the dynamic viscosity of the hydrocarbon–air combustion products *μ*_prod_ differed little from that of air, with a minor correction depending on the equivalence ratio *ϕ* [61], and is written as

A 3 $$\mu_{\mathrm{prod}}=\frac{\mu_{\mathrm{air}}}{1+0.027\phi },\;\mathrm{for}\;T\in [500,4000]\;K,\;\phi \in [0,4].$$

The exponential component in propagating waves can be changed to

A 4 $$\exp(\pm ik^{\pm }\Delta x)=\exp(\mp \tau^{\pm }(s+\Lambda s^{1/2})),$$

where $\tau^{\pm }=\Delta x/(\bar{c}\pm \bar{u})$ and Δ*x* is the path length of the travelling acoustic wave.

**(b) The expressions of $M_{n}$**

$$\begin{array}{rl} M_{1} & =1+\frac{S_{1}}{S_{2}}\left({\bar{M}}_{1}(2+{\bar{M}}_{1})-\frac{{\bar{c}}_{2}}{{\bar{c}}_{1}}{\bar{M}}_{2}(1+{\bar{M}}_{1})\right)\;M_{3}=1+{\bar{M}}_{2}\\ M_{2} & =1-\frac{S_{1}}{S_{2}}\left({\bar{M}}_{1}(2-{\bar{M}}_{1})-\frac{{\bar{c}}_{2}}{{\bar{c}}_{1}}{\bar{M}}_{2}(1-{\bar{M}}_{1})\right)\;M_{4}=1-{\bar{M}}_{2}\\ M_{5} & =1+\gamma {\bar{M}}_{1}+(\gamma -1){\bar{M}}_{1}^{2}\;M_{7}=1+\gamma {\bar{M}}_{2}+(\gamma -1){\bar{M}}_{2}^{2}\\ M_{6} & =1-\gamma {\bar{M}}_{1}+(\gamma -1){\bar{M}}_{1}^{2}\;M_{8}=1-\gamma {\bar{M}}_{2}+(\gamma -1){\bar{M}}_{2}^{2}, \end{array}$$

where $M=u/\bar{c}$ is the Mach number.
